# Effects of Smoking and Smoking Cessation on Life Expectancy in an Elderly Population in Beijing, China, 1992–2000: An 8-Year Follow-up Study

**DOI:** 10.2188/jea.JE20110001

**Published:** 2011-09-05

**Authors:** Xiaobing Tian, Zhe Tang, Jingmei Jiang, Xianghua Fang, Xiaoguang Wu, Wei Han, Shaochen Guan, Hongjun Liu, Lijun Diao, Fei Sun

**Affiliations:** 1 Department of Epidemiology & Social Medicine, Xuanwu Hospital, Capital Medical University, Beijing, China; 2 Department of Epidemiology and Statistics, Institute of Basic Medical Sciences Chinese Academy of Medical Sciences, School of Basic Medicine Peking Union Medical College, Beijing, China

**Keywords:** smoking, smoking cessation, life expectancy, active life expectancy, elderly population, discrete-time hazard model

## Abstract

**Background:**

We assessed the effects of smoking and smoking cessation on life expectancy and active life expectancy among persons aged 55 years or older in Beijing.

**Methods:**

This study included 1593 men and 1664 women who participated in the Beijing Longitudinal Study of Aging, which commenced in 1992 and had 4 survey waves up to year 2000. An abridged life table was used to estimate life expectancy, in which age-specific mortality and age-specific disability rates were adjusted by using a discrete-time hazard model to control confounders.

**Results:**

The mean ages (SD) for men and women were 70.1 (9.25) and 70.2 (8.72) years, respectively; mortality and disability rates during follow-up were 34.7% and 8.0%, respectively. In both sexes, never smokers had the highest life expectancy and active life expectancy across ages, as compared with current and former smokers. Current heavy smokers had a shorter life expectancy and a shorter active life expectancy than light smokers. Among former smokers, male long-term quitters had a longer life expectancy and longer active life expectancy than short-term quitters, but this was not the case in women.

**Conclusions:**

Older adults remain at higher risk of mortality and morbidity from smoking and can expect to live a longer and healthier life after smoking cessation.

## INTRODUCTION

A number of studies have reported that cigarette smoking shortens life expectancy (LE) and decreases quality of life.^1^^–^^6^ However, most of these studies focused on young or middle-aged populations,^7^ and some other studies grouped older adults with widely varying ages in a single category.^2^^,^^5^ Consequently, it is unclear whether smokers who survive to advanced age remain at a high risk of death from smoking and, if so, what the magnitude of the risk is. Moreover, although LE and active life expectancy (ALE) have been widely used to quantify the adverse effects of smoking,^1^^,^^8^^,^^9^ these descriptive measurements can lead to biased estimates for older adults—for whom various mortality and morbidity risks are present—due to the absence of adjustment for confounding variables.^10^^,^^11^ In addition, the beneficial effects of smoking cessation remain controversial. Some studies showed that mortality risk 10 to 15 years after smoking cessation was comparable to that in never smokers^12^; however, some researchers have argued that, from a biological perspective, the extent of the benefit depends on the reversibility of the relevant disease processes at the time of cessation.^13^ For older persons who have smoked for years, the cumulative harmful effects might be quite considerable and possibly not quickly eradicable. This remains to be elucidated by further studies of potential interventions.

The objective of this study was to systematically assess the adverse effects of smoking and the beneficial effects of smoking cessation among elderly adults in terms of life expectancy and active life expectancy. A discrete-time hazard model was used to control confounders while calculating age-specific mortality to construct life tables.^14^^,^^15^ Death or new disability during follow-up was used as a combined outcome in the estimation of ALEs. Data for these analyses came from the Beijing Longitudinal Study of Aging (BLSA), a prospective cohort of 3257 male and female residents of Beijing, China, who were aged 55 years or older in 1992–2000.

## METHODS

### Data collection during follow-up

The BLSA started in Beijing, the capital city of China, in 1992. Beijing consists of 18 administrative districts that were divided into 3 categories according to the degree of urbanization and economic status: there were 8 main cities, 5 suburbs, and 5 extended suburbs. To select a representative sample whose geographic distribution, economic status, age, and education were similar to those of the Beijing population older than 55 years, a multi-step stratified random sampling method was used during cohort establishment. First, 1 administrative district was chosen from each category, with the restriction that age and education level must be parallel with those of the overall population in Beijing and that economic development was at the average level for the category. The districts selected were Xuanwu district (urban), Daxing county (suburb), and Huairou county (extended suburb). Second, specific neighborhoods were randomly selected from within these districts based on age distribution, sex distribution, and educational level of the population, in order to be representative of these distributions in the district overall. For Xuanwu district, 2 of 9 neighborhood units were selected. For Daxing, 2 of 27 villages were identified. For Huairou, 1 of 21 villages was chosen. Third, a predetermined number of subjects were selected from these neighborhood units and villages using a systematic sampling method.

After these sampling steps, the total number of subjects selected for this study was 3579 (65.6% were from urban, 21.1% from suburban, and 13.3% from extended suburban districts). Among the eligible participants, 3257 agreed to participate in the survey (response rate 91%). At the initial interview, and again after 2, 5, and 8 years of follow-up, all participants in the cohort received a questionnaire encompassing a wide range of psychosocial and health-related issues, including socioeconomic status, activities of daily living, physical exercise, and self-evaluated health status and medical condition. The questionnaire was completed at home and verified by trained senior medical students, who also completed the questionnaire on behalf of illiterate participants. Informed consent was obtained from all participants at baseline.

In the present study, suburban and extended suburban communities were combined into 1 group (ie, rural sites) for analyses requiring comparison with urban elderly adults. The BLSA data were stratified by sex and age. Age was categorized in 5-year age categories from 55 through 80 years plus an age group containing persons older than 80, which resulted in 6 age groups of approximately equal size. The distribution of educational categories in the final dataset was consistent with that of the older population of Beijing obtained from the Fourth National Census Data of China.^16^

Smoking status was coded as current, former, or never smoking on the basis of responses to 2 questions at the initial interview: “Do you regularly smoke now?” and “Were you a regular smoker in the past?” Current and former smokers also provided information on average number of cigarettes smoked per day and number of years smoked. Current smokers were subdivided into light smokers (<20 cigarettes per day) and heavy smokers (≥20 cigarettes per day). Former smokers were also further subdivided into 2 subgroups by quitting time: long-term quitters were those who had quit at least 5 years earlier, and short-term quitters were those who had quit less than 5 years earlier. A never smoker was defined as a person who had never smoked or had only smoked infrequently at a young age.

At baseline and each follow up-interview, participants were asked to report their ability to perform several basic daily activities, such as walking across a small room, moving from bed to chair, bathing, dressing, eating, and grooming. A self-reported need for help or inability to perform any of these activities was considered as a state of disability.

### Statistical methods

#### Life expectancy calculation

An abridged life table (proposed by Sulivan et al) was used to calculate LE.^17^ In the construction of the life table, we introduced the correction coefficient (*z*)—which was determined by the effect and distribution of confounders—for adjustment of age-specific mortality (*m_x_*). Confounders were screened by change-in-estimation, and their effects were quantified by using a discrete-time hazard model. The details of the calculation are illustrated by the following 3 steps.

(1) *Construction of discrete time-hazard model.* We constructed a model with all variables as explanatory variables, as shown in equation (1).

$$\log it\left(\frac{p_{ij}}{1-p_{ij}}\right)=\alpha_{1}D_{1ij}+\alpha_{2}D_{2ij}+\cdots +\alpha_{J}D_{Jij}+\beta_{smoking\;status}+\beta_{age\;group}+\beta_{1}x_{1ij}+\beta_{2}x_{2ij}+\cdots +\beta_{p}x_{pij}$$ (1)

where *p*_ij_ denotes the probability that person *i* with covariates *j* will experience death during a follow-up period, conditional on their event-free survival up to the start of time interval j. [D_1ij_, D_2ij_,…, D_Jij_] are a series of dummy variables indexing follow-up periods, [α_1_, α_2_,…, α_J_] are the intercept parameters, and [β_1_, β_2_,…, β_J_] are the slope parameters that describe the effect of the predictors on the baseline model. Specifically, smoking status was defined using dummy variables, with never smokers as reference.

(2) *Screening of confounders.* We removed the variable with the smallest Wald Chisq value from the original model. We repeated this procedure in the just-derived models until the final model included only age group and smoking status. At each step, the difference in the coefficients for smoking status between the original model and successive model was calculated. Variables were defined as confounders if there was a change greater than 10%. Six of 10 variables were selected as explanatory variables according to these change-in-estimation criteria, which were marital status, education, district, alcohol consumption (in men only), self-reported health status, and physical disabilities. The other 4 variables, namely, occupation (present or past), annual family income, satisfaction with medical care, and satisfaction with daily living were excluded. The validity of the final model with selected covariates was evaluated using the Hosmer-Lemeshow goodness-of-fit test, and the model was considered to be valid if the corresponding *P*-value was greater than 0.05. Based on this criterion, all models were shown to be valid (see the results below and Tables 3 and 4).

(3) *Calculation of adjusted age-specific mortality.* We multiplied the corresponding coefficients in the model by the proportions of individuals (according to smoking status and sex) in each specific confounder (Table 2). Next, we added the product to the intercept and to the value of the age coefficient for the group to obtain *z*.^14^ Adjusted age-specific mortality was calculated by using equation (2):

$$m_{x}{}^{\prime }=\frac{1}{1+e^{-z}}$$ (2)

where *x* is a specific age group, *z* is the correction coefficient for the age group of interest and smoking status of interest, and *m_x_*′ is the adjusted age-specific mortality.

#### Calculation of active life expectancy

For calculating ALE, the outcome variable was defined as death or new disability (*n* = 262). Participants who reported a disability in daily living (*n* = 268) at the baseline interview were excluded from the study population. The procedure for estimation was the same as that used for LE.

All statistical analyses were performed using the SAS statistical software (Version 9.2).

## RESULTS

A total of 3257 persons were interviewed at baseline; 62.7% were urban residents and 37.3% were rural residents. During follow up, 1130 (34.7%) died, 262 (8.0%) reported new disabilities, 1705 (52.3%) were right-censored, and 160 (4.9%) were lost to follow-up (Table 1).

**Table 1.** Number of participants in each follow-up survey of Beijing adults aged 55 years or older, 1992–2000

Interview time	1992	……	1994	……	1997	……	2000
Invited (survivors of original cohort)	3257		2703		2143		1705
Died between interviews		363		436		331	
Lost to follow-up		72		47		41	

Ultimately, 1593 (49.1%) men and 1664 (50.9%) women were included in the analysis. The mean ages (SD) for men and women were 70.1 (9.25) and 70.2 (8.72) years, respectively. In comparison with women, there was a lower proportion of never smokers among men (74.7% vs. 33.2%), and higher proportions of former smokers (9.8% vs. 23.3%) and current smokers (15.5% vs. 43.5%); the differences between sexes were statistically significant (*P* < 0.001).

Table 2 shows the distribution of smoking status by sex and covariate. Approximately 75% of men and 50% of women were currently married, and more than 21% of men and 70% of women were illiterate. Among men, 80% of never smokers did not consume alcohol, while 54% of current heavy smokers reported drinking alcohol. Among men, 43% of short-term quitters were in bad health, as were 61% of female short-term quitters. These proportions were larger than those for other smoking statuses. Also, among short-term quitters, 13% of men and 14% of women reported disabilities at baseline.

**Table 2.** Characteristics of participants at baseline, by sex and smoking status

Categories	Smoking status									

	Never	Former		Current		Never	Former		Current	
			
		>5 yrs	≤5 yrs	<20 yrs	≥20 yrs		>5 yrs	≤5 yrs	<20 yrs	≥20 yrs

	Males					Females				
Mean age, yrs	70.8	72.7	70.9	70.1	65.8	69.7	74.7	69.8	69.9	71.5
Marital status (%)										
Currently married	76.2	77.3	77.0	75.7	79.0	57.7	37.1	44.9	51.1	38.5
Single^a^	23.8	22.7	23.0	24.3	21.0	42.3	62.9	55.1	48.9	61.5
Education (%)										
Secondary or higher	25.3	19.8	11.0	14.4	7.4	9.5	2.1	0.0	2.2	3.9
Primary	47.8	58.3	53.0	52.2	57.6	20.5	29.9	20.4	20.8	19.2
Illiterate	26.9	21.9	36.0	33.4	34.9	70.0	68.0	79.6	77.0	76.9
Community (%)										
Urban	70.7	83.1	59.0	58.8	52.8	63.8	74.2	63.3	71.4	80.8
Rural	29.3	16.9	41.0	41.2	47.2	36.2	25.8	36.7	28.6	19.2
Consumption of alcohol (%)										
No	80.2	69.8	67.0	50.2	45.9	96.0	89.7	89.8	84.0	84.6
Yes	19.8	30.2	33.0	49.8	54.1	4.0	10.3	10.2	16.0	15.4
Self-reported health status (%)										
Good	63.1	63.6	53.0	59.7	63.8	53.8	55.7	38.8	55.4	65.4
Not good	31.8	33.1	43.0	36.9	33.6	42.8	41.2	61.2	42.4	30.8
NA	5.1	3.3	4.0	3.4	2.6	3.4	3.1	0.0	2.2	3.8
Disabled^b^ (%)										
No	92.4	90.1	87.0	95.5	97.8	90.0	89.7	85.7	93.1	96.2
yes	7.6	9.9	13.0	4.5	2.2	10.0	10.3	14.3	6.9	3.8

^a^Includes divorced and widowed subjects

^b^no: no self-reported disability at baseline; yes: self-reported disability at baseline

Sex-specific life expectancy at attained ages is shown in Table 3. In general, never smokers had the highest LE, as compared with former and current smokers, and current light smokers lived longer than heavy smokers. Among men, long-term quitters had a higher LE than short-term quitters; however, the opposite was true in women. The graded difference, which depended on intensity and cessation duration, remained constant across all age groups. Women had a higher LE than did men in corresponding groups, except among heavy smokers and long-term quitters.

**Table 3.** Life expectancy of Beijing adults aged 55 years or older, by sex and smoking status (1992–2000)

Age, yrs	Smoking status				

	Never	Former		Current	
		
	Never (95% CI)	>5 yrs (95% CI)	≤5 yrs (95% CI)	<20 yrs (95% CI)	≥20 yrs (95% CI)
*Males*^a^					
55–59	25.5 (24.8–25.6)	22.5 (21.9–23.1)	20.3 (19.4–21.1)	22.5 (22.1–22.9)	21.0 (20.3–21.7)
60–64	21.8 (21.4–22.2)	19.2 (18.7–19.8)	17.2 (16.4–18.0)	19.2 (18.8–19.7)	17.9 (17.2–18.6)
65–69	18.3 (17.9–18.7)	15.9 (15.4–16.5)	14.0 (13.2–14.8)	15.9 (15.5–16.4)	14.7 (13.9–15.4)
70–74	15.2 (14.8–15.6)	12.9 (12.4–13.5)	11.2 (10.0–12.0)	13.0 (12.5–13.4)	11.8 (10.9–12.7)
75–79	12.9 (12.4–13.4)	10.8 (10.2–11.5)	9.2 (8.2–10.2)	10.8 (10.3–11.4)	9.7 (8.7–10.8)
80–	11.2 (10.6–11.8)	9.2 (8.4–9.9)	7.6 (6.4–8.9)	9.2 (8.5–9.9)	8.1 (6.8–9.5)
*Females*^b^					
55–59	26.6 (26.3–26.9)	20.9 (19.9–21.9)	23.8 (22.4–25.2)	24.8 (24.2–25.5)	17.8 (16.1–19.4)
60–64	22.5 (22.2–22.8)	17.0 (16.2–17.1)	19.8 (18.4–21.2)	20.8 (20.1–21.4)	14.1 (12.5–15.7)
65–69	19.1 (18.8–19.1)	14.0 (13.2–14.8)	16.6 (15.1–18.1)	17.5 (16.8–18.2)	11.4 (9.9–12.9)
70–74	16.1 (15.7–16.4)	11.3 (10.5–12.0)	13.7 (12.1–15.8)	14.5 (13.8–15.2)	9.0 (7.4–10.6)
75–79	13.7 (13.3–14.0)	9.2 (8.4–9.9)	11.3 (9.6–13.3)	12.2 (11.4–13.0)	7.1 (5.7–8.6)
80–	11.8 (11.4–12.2)	7.5 (6.7–8.4)	9.6 (7.3–12.0)	10.4 (9.4–11.4)	5.6 (3.5–7.7)

Hosmer-Lemeshow goodness-of-fit test for sex-specific model used to calculate correction coefficient:

^a^Males: *x^2^* = 5.81, *P* = 0.67

^b^Females: *x^2^* = 12.16, *P* = 0.14

**Figure 1.**
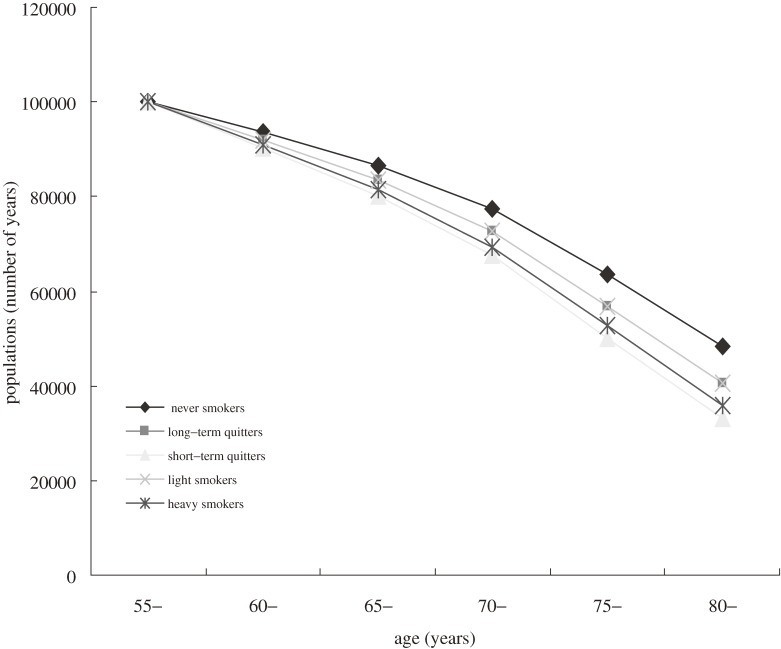
Survival curves for a population of 100 000 men, starting from age 55 years.

**Figure 2.**
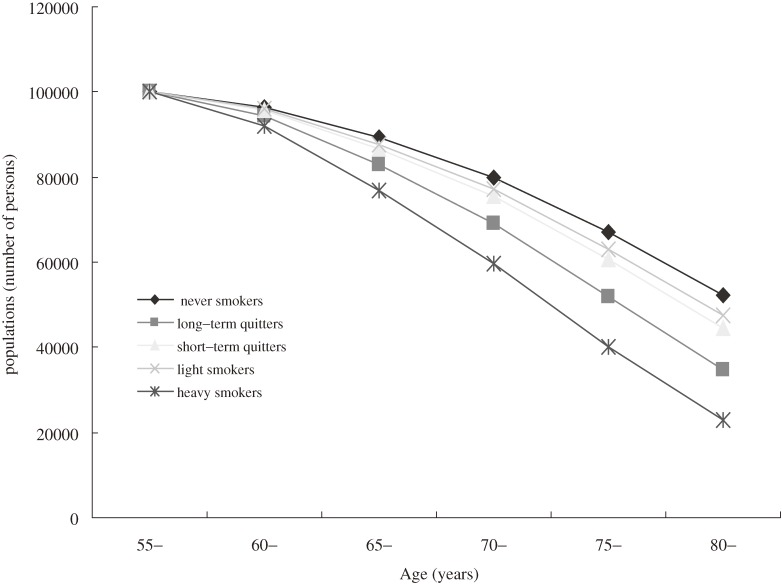
Survival curves for a population of 100 000 women, starting from age 55 years.

Survival curves (commencing at age 55 years) were plotted by sex according to smoking status (Figures 1, 2). The y-axis was standardized to 100 000 persons at age 55 for all smoking status groups, the death rates of which were followed to old age. Overall, survival curves gradually diverged with aging. Never smokers had the best survival in both sexes. In men, short-term quitters had the lowest survival, whereas in women, heavy smokers had the lowest survival. In addition, the survival curves were more disparate in women.

Sex-specific ALEs were also calculated according to smoking status (Table 4). In general, active life expectancies were lower than corresponding life expectancies. Similar trends in the variation and extent of discrepancies between groups were observed.

**Table 4.** Active life expectancy of Beijing adults aged 55 years or older, by sex and smoking status (1992–2000)

Age, yrs	Smoking status				

	Never	Former		Current	
		
	Never (95% CI)	>5 yrs (95% CI)	≤5 yrs (95% CI)	<20 yrs (95% CI)	≥20 (95% CI)
*Males*^a^					
55–59	23.1 (22.7–23.4)	20.2 (19.2–21.1)	18.9 (18.1–19.7)	21.2 (20.8–21.6)	21.1 (20.4–21.7)
60–64	19.7 (19.4–20.1)	17.1 (16.1–18.1)	16.0 (15.2–16.7)	18.0 (17.6–18.4)	17.9 (17.2–18.6)
65–69	16.4 (16.0–16.8)	14.0 (12.9–15.1)	13.0 (12.2–13.7)	14.8 (14.4–15.2)	14.7 (13.9–15.5)
70–74	13.2 (12.8–13.6)	11.0 (9.7–12.3)	10.1 (9.3–10.9)	11.8 (11.3–12.2)	11.7 (10.8–12.5)
75–79	10.8 (10.3–11.2)	8.7 (7.1–10.3)	7.9 (7.0–8.8)	9.4 (8.9–9.9)	9.3 (8.3–10.4)
80–	8.8 (8.3–9.3)	6.9 (6.2–7.5)	6.1 (5.0–7.3)	7.5 (6.9–8.1)	7.4 (6.1–8.7)
*Females*^b^					
55–59	24.9 (24.7–25.2)	20.9 (19.7–22.0)	22.2 (21.3–23.1)	24.7 (24.0–25.3)	20.2 (18.5–21.8)
60–64	20.8 (20.5–21.0)	16.9 (15.8–18.0)	18.2 (17.3–19.1)	20.5 (19.9–21.2)	16.3 (14.7–17.9)
65–69	17.2 (16.9–17.5)	13.6 (12.6–14.7)	14.8 (13.9–15.7)	17.0 (16.3–17.7)	13.2 (11.6–14.8)
70–74	14.1 (13.8–14.4)	10.8 (9.8–11.8)	11.8 (10.9–12.8)	13.9 (13.2–14.6)	10.1 (8.4–11.7)
75–79	11.5 (11.2–11.8)	8.5 (7.6–9.4)	9.4 (8.4–10.5)	11.3 (10.5–12.1)	7.8 (6.2–9.4)
80–	9.5 (9.1–9.9)	6.7 (5.7–7.7)	7.5 (6.3–8.8)	9.3 (8.4–10.3)	6.1 (3.9–8.3)

Hosmer-Lemeshow goodness-of -fit test for sex-specific model used to calculate correction coefficient:

^a^Males: *x^2^* = 4.87, *P* = 0.77

^b^Females: *x^2^* = 9.36, *P* = 0.31

Table 5 displays reduced life expectancy and active life expectancy for former and current smokers, with never smokers as reference. In both men and women, life reduction decreased with aging, and heavy smokers had greater reductions than light smokers. In men, short-term quitters had the greatest life reduction, while in women, current heavy smokers had the greatest reduction in life expectancy. Similar variation between groups was also observed with respect to ALE reduction. Notably, among men, long-term quitters and current light smokers had a similar reduction in life expectancy, and short-term quitters had a greater reduction in life expectancy than current light smokers; however, in women, both long-term and short-term quitters had a greater reduction in life expectancy than current light smokers.

**Table 5.** Reduced life expectancy and active life expectancy of Beijing adults aged 55 years or older, by sex and smoking status (1992–2000)

Category		Smoking status				

		Never	Former		Current	
	
			>5 yrs	≤5 yrs	<20 yrs	≥20 yrs
*Reduced life expectancy*						
55–64	Males	25.2	−2.7	−4.9	−2.7	−4.2
	Females	26.6	−5.7	−2.8	−1.8	−8.8
65–74	Males	18.3	−2.4	−4.3	−2.4	−3.6
	Females	19.1	−5.1	−2.5	−1.6	−7.7
75–	Males	12.9	−2.1	−3.7	−2.1	−3.2
	Females	13.7	−4.5	−2.3	−1.5	−6.6
*Reduced active life expectancy*						
55–64	Males	23.1	−2.9	−4.2	−1.9	−2.0
	Females	24.9	−4.0	−2.7	−0.2	−4.7
65–74	Males	16.4	−2.4	−3.4	−1.6	−1.7
	Females	17.2	−3.6	−2.4	−0.2	−4.0
75–	Males	10.8	−2.1	−2.9	−1.4	−1.5
	Females	11.5	−3.0	−2.1	−0.2	−3.7

## DISCUSSION

In this 8-year follow-up study, we estimated sex-specific life expectancy and active life expectancy for individuals aged 55 years or older according to smoking status. We found a graded difference in LE and ALE with respect to smoking status (ie, never smokers and current light and heavy smokers) across ages in both sexes, demonstrating that older adults were still at high risk of mortality and morbidity from smoking. Our results also showed that male long-term quitters had lower reductions in LE and ALE than short-term quitters, suggesting that older male smokers could expect to benefit from smoking cessation, although this was not the case in women.

In China, 2 nationwide smoking prevalence surveys have been conducted, in 1984 and 1996,^18^^,^^19^ both of which revealed an increasing prevalence of smoking, rising mean daily cigarette consumption, and declining age of smoking initiation. These studies led to the prediction that tobacco would eventually have large health consequences in China. A population-based retrospective study by Liu et al^20^ in 1989 and a prospective cohort study by Yuan et al in 1984^21^ provided further evidence of the devastating impact of smoking on lung cancer, respiratory disease, and heart disease in terms of proportional mortality and relative risks. As a result of these studies, tobacco control was initiated with an emphasis on preventing young people from starting smoking and encouraging smoking cessation at a young age. Our findings that older adults remains at high risk from smoking and that they can still benefit from smoking cessation highlight the importance of tobacco control among this population.

A reduction in life expectancy due to smoking has been shown in previous studies. In Copenhagen, the reduction in the life expectancy of heavy smokers aged 35 years, as estimated in a population-based study, was 9.2 years in men and 9.4 years in women.^1^ In the Framingham Heart study, the difference in life expectancy between a 50-year-old current smoker and never smoker was 8.66 years in men and 7.59 years in women, based on data from a follow-up study.^22^ Our estimates confirm the overall conclusion of the literature, namely, that the life expectancy of smokers is less than that of never smokers. However, the extent of lost life expectancy was less in our study, especially for men, possibly because of differences between studies in participant age and study setting and methodology.

During the period before 1990, when the adverse effects of smoking were less well known among the general population of China, smoking was so prevalent—approximately 70% of adult men were smokers—that non-smoking was usually related to existing health problems.^18^ Among nonsmokers, 53.5% were passive smokers. Moreover, a special group of so-called “social smokers”, ie, persons who smoke only when they are in certain social settings, always regarded themselves as nonsmokers.^23^ Therefore, the reference group (never smokers) might have included some passive smokers and irregular smokers, which would have diluted the observed difference. In contrast, estimates for women had no such problems because smoking was not socially accepted for women at that time. Our results are more similar to those found in Japan,^24^ where a study showed that the difference in life expectancy between current smokers and never smokers was 3 years for 55-year-old men who were recruited in 1980, when the smoking prevalence was 62.9%, which is similar to the 70% prevalence in China.

The discrepancy in findings on life expectancy might also be due to methodological differences in calculating age-specific mortality. We used adjusted mortality to construct life tables by using a discrete-hazard model. Some risk factors, which themselves have effects on morbidity and mortality, could lead to overestimation of risk.^10^^,^^25^ Roger et al^14^ found that the adjusted odds ratios were reduced by 13%, 17%, and 29% for light smokers, moderate smokers, and heavy smokers, respectively, as compared with unadjusted estimates. Streppel et al^8^ reported that the between-group difference in life expectancy classified by smoking status was reduced after controlling for potential confounders. In light of these findings, we controlled confounders such as education, marital status, and community and alcohol consumption in estimating age-specific mortality rate. Unlike other studies, we did not include factors such as daily physical exercise and psychological factors as explanatory variables, as they might be outcomes of disability.

Our findings revealed that both long-term and short-term quitters had a substantial loss in life expectancy. This seems incompatible with other studies, which showed health benefits increased proportionally to the number of years since smoking cessation.^26^ However, some numerical results must be interpreted in the context of study population, design, and/or outcome.^27^ Our results might be population-specific, as they could reflect the continuous and cumulative harmful effects of tobacco use on mortality and morbidity among elderly populations. In our study, short-term cessation meant that a person stopped smoking at a relatively advanced age (age 50 years or older). Among long-term quitters, only 18.9% had stopped smoking for more than 10 years (not presented in results), which implies that they had smoked for a long period of time, as was shown in some studies. In such individuals, the adverse effects of smoking are unlikely to be quickly reversed.^13^ Moreover, in China, no scaled-up intervention has been targeted at older adults, and consultation from medical professionals on smoking cessation was unlikely because more than 56% of doctors were smokers at the time of the survey.^18^ Thus, the logical explanation for smoking cessation at a relatively advanced age is that bad health compelled participants to stop.

Other studies also found an increased risk in former smokers. Østbye et al reported greater loss of life expectancy in those who had stopped smoking less than 3 years earlier in comparison with light smokers,^28^ and Leffondré et al found that ex-smokers had a higher risk of lung cancer than did current smokers.^27^ Results from this and other studies, however, do not negate the impact of smoking cessation during old age. In a large-scale cohort study in Japan, the increase in life expectancy of former male smokers who quit smoking before the ages of 40, 50, 60, and 70 years was 4.8, 3.7, 1.6, and 0.5 years, respectively.^3^ A British doctor cohort study revealed that those who stopped smoking at 60 years of age could expect to gain 3 years of life.^9^ Our results also showed that loss of life expectancy decreased with increasing duration of smoking cessation in men, suggesting that it was never too late to stop smoking.

Although quality of life is a commonly used concept, it has no universally accepted definition. In our definition we stressed daily physical activity because it is a primary self-evaluated index for most Chinese older adults and can be easily and objectively observed. The graded difference associated with smoking intensity and duration since cessation can also be reflected by ALE, which indicated that non-smokers who have a longer life expectancy, in spite of subsequent longer period of disability exposure, still enjoyed a longer ALE. Previous studies of the effects of smoking found that smoking increases the risks of a wide range of chronic disease,^21^ which were significantly associated with mortality as well as disability. This implies that the observed reduction in active life expectancy is biologically plausible.

Smoking among women has been a growing concern. Previous studies indicated that female smokers are more susceptible to a number of fatal diseases and that they have more difficulty quitting than men.^29^^,^^30^ Our conclusion was consistent: female heavy smokers experienced a greater reduction in life expectancy as compared with their male counterparts. However, female long-term quitters had a lower LE/ALE than female short-term quitters, which seems counterintuitive. The probable reason for the lower LE/ALE among the former group is that smoking cessation is often triggered by the presence of life-threatening illness. The presence of such medical conditions reverses the negative causal association between duration of smoking cessation and mortality rate. Moreover, the interval from initiation of smoking cessation (disease manifestation) to death is determined by the natural course of the fatal disease, which would increase the mortality rate among long-term quitters (defined as >5 years in the present study). Therefore, this result does not exclude the necessity of encouraging elderly women to quit smoking, but rather highlights the importance of increasing health care provided to elderly women who attempt to quit smoking.

There were some limitations in our study. Overall, 4.9% of subjects were lost to follow up. Although this was lower than values in some previous studies, we cannot exclude the possibility that these subjects were at higher risk of death or disability in comparison with the remaining subjects or that their loss to follow-up was related to their smoking status, which would increase the potential for selection bias. Misclassification of smokers and never-smokers was also a possibility. If individuals classified as smokers at the time of the baseline survey subsequently quit, this could have contributed to a lower mortality rate in the smoker group because of their improved health. Another source of possible misclassification was the “social smokers” mentioned above. There were few female heavy smokers. Thus, the estimates in women had wide confidence intervals, which reduces the reliability of the estimates. There is also a possibility of residual confounding. Thus, we think that a priori knowledge, possibly based on previous analyses, is necessary for controlling potential confounders in future studies.

In conclusion, our study suggests that never smokers live longer and healthier lives and that smoking cessation is beneficial even among older adults.

## References

[1] Prescott E , Osler M , Hein HO , Borch-Johnsen K , Schnohr P , Vestbo J Life expectancy in Danish women and men related to smoking habits: smoking may affect women more . J Epidemiol Community Health. 1998;52(2):131–2 10.1136/jech.52.2.131 9578864 PMC1756671

[2] Peto R , Lopez AD , Boreham J , Thun M , Heath C Jr , Doll R Mortality from smoking worldwide . Br Med Bull. 1996;52(1):12–21 8746293 10.1093/oxfordjournals.bmb.a011519

[3] Ozasa K , Katanoda K , Tamakoshi A , Sato H , Tajima K , Suzuki T , Reduced life expectancy due to smoking in large-scale cohort studies in Japan . J Epidemiol. 2008;18(3):111–8 10.2188/jea.JE2007416 18480591 PMC4771605

[4] Jiang J , Liu B , Sitas F , Li J , Zeng X , Han W , Smoking-attributable deaths and potential years of life lost from a large, representative study in China . Tob Control. 2010;19(1):7–12 10.1136/tc.2009.031245 19748886

[5] Corrêa PC , Barreto SM , Passos VM Smoking-attributable mortality and years of potential life lost in 16 Brazilian capitals, 2003: a prevalence-based study . BMC Public Health. 2009;9:206 10.1186/1471-2458-9-206 19558658 PMC2711948

[6] Sarna L , Bialous SA , Cooley ME , Jun HJ , Feskanich D Impact of smoking and smoking cessation on health-related quality of life in women in the Nurses’ Health Study . Qual Life Res. 2008;17(10):1217–27 10.1007/s11136-008-9404-8 18931942 PMC4729379

[7] Richardson L , Hemsing N , Greaves L , Assanand S , Allen P , McCullough L , Preventing smoking in young people: a systematic review of the impact of access interventions . Int J Environ Res Public Health. 2009;6(4):1485–514 10.3390/ijerph6041485 19440530 PMC2681197

[8] Streppel MT , Boshuizen HC , Ocké MC , Kok FJ , Kromhout D Mortality and life expectancy in relation to long-term cigarette, cigar and pipe smoking: the Zutphen Study . Tob Control. 2007;16(2):107–13 10.1136/tc.2006.017715 17400948 PMC2598467

[9] Doll R , Peto R , Boreham J , Sutherland I Mortality in relation to smoking: 50 years’ observations on male British doctors . BMJ. 2004;328(7455):1519 10.1136/bmj.38142.554479.AE 15213107 PMC437139

[10] Cristia JP Rising mortality and life expectancy differentials by lifetime earnings in the United States . J Health Econ. 2009;28(5):984–95 10.1016/j.jhealeco.2009.06.003 19616863

[11] Carter KN , Blakely T , Soeberg M Trends in survival and life expectancy by ethnicity, income and smoking in New Zealand: 1980s to 2000s . N Z Med J. 2010;123(1320):13–24 20720599

[12] Pelkonen M , Notkola IL , Tukiainen H , Tervahauta M , Tuomilehto J , Nissinen A Smoking cessation, decline in pulmonary function and total mortality: a 30 year follow up study among the Finnish cohorts of the Seven Countries Study . Thorax. 2001;56(9):703–7 10.1136/thorax.56.9.703 11514691 PMC1746131

[13] Appel DW , Aldrich TK Smoking cessation in the elderly . Clin Geriatr Med. 2003;19(1):77–100 10.1016/S0749-0690(02)00053-8 12735116

[14] Rogers RG, Hummer RA, Krueger PA, Pampel FC. Combining Prevalence and Mortality Risk Rates: The Case of Cigarette Smoking. In: University of Colorado; 2002. p. 15–9. 10.1111/j.1728-4457.2005.00065.x PMC4098763 25035524

[15] Richardson DB Discrete time hazards models for occupational and environmental cohort analyses . Occup Environ Med. 2010;67(1):67–71 10.1136/oem.2008.044834 20029026

[16] China NBOS. Statistical communiques on the Fourth National Census in 1990 (4th): http://www.stats.gov.cn/tjgb/rkpcgb/qgrkpcgb/t20020404_16774.htm In; 1990.

[17] Jagger C, Cox B, Le Roy S. Health Expectancy Calculation by the Sullivan Method: A Practical Guide. EHEMU Technical Report September 2006. EHEMU; 2007.

[18] Yang G , Fan L , Tan J , Qi G , Zhang Y , Samet JM , Smoking in China: findings of the 1996 National Prevalence Survey . JAMA. 1999;282(13):1247–53 10.1001/jama.282.13.1247 10517427

[19] Weng XZ , Hong ZG , Chen DY Smoking prevalence in Chinese aged 15 and above . Chin Med J (Engl). 1987;100(11):886–92 3130228

[20] Liu BQ , Peto R , Chen ZM , Boreham J , Wu YP , Li JY , Emerging tobacco hazards in China: 1. Retrospective proportional mortality study of one million deaths . BMJ. 1998;317(7170):1411–22 9822393 10.1136/bmj.317.7170.1411 PMC28719

[21] Yuan JM , Ross RK , Wang XL , Gao YT , Henderson BE , Yu MC Morbidity and mortality in relation to cigarette smoking in Shanghai, China. A prospective male cohort study . JAMA. 1996;275(21):1646–50 10.1001/jama.275.21.1646 8637137

[22] Mamun AA , Peeters A , Barendregt J , Willekens F , Nusselder W , Bonneux L ; NEDCOM, The Netherlands Epidermiology and Demography Compression of Morbidity Research Group Smoking decreases the duration of life lived with and without cardiovascular disease: a life course analysis of the Framingham Heart Study . Eur Heart J. 2004;25(5):409–15 10.1016/j.ehj.2003.12.015 15033253

[23] Pan Z , Hu D Hierarchical linear modelling of smoking prevalence and frequency in China between 1991 and 2004 . Health Policy Plan. 2008;23(2):118–24 10.1093/heapol/czm043 17998239

[24] Murakami Y , Ueshima H , Okamura T , Kadowaki T , Hozawa A , Kita Y , Life expectancy among Japanese of different smoking status in Japan: NIPPON DATA80 . J Epidemiol. 2007;17(2):31–7 10.2188/jea.17.31 17420610 PMC7058458

[25] Pednekar MS , Gupta PC , Hebert JR , Hakama M Joint effects of tobacco use and body mass on all-cause mortality in Mumbai, India: results from a population-based cohort study . Am J Epidemiol. 2008;167(3):330–40 10.1093/aje/kwm293 17989059

[26] Samet JM The health benefits of smoking cessation . Med Clin North Am. 1992;76(2):399–414 1548968 10.1016/s0025-7125(16)30359-5

[27] Neuner B , Wellmann J , Dasch B , Behrens T , Claes B , Dietzel M , Modeling smoking history: a comparison of different approaches in the MARS study on age-related maculopathy . Ann Epidemiol. 2007;17(8):615–21 10.1016/j.annepidem.2007.03.005 17531503

[28] Østbye T , Taylor DH The effect of smoking on years of healthy life (YHL) lost among middle-aged and older Americans . Health Serv Res. 2004;39(3):531–52 10.1111/j.1475-6773.2004.00243.x 15149477 PMC1361023

[29] Benowitz NL Clinical pharmacology of nicotine: implications for understanding, preventing, and treating tobacco addiction . Clin Pharmacol Ther. 2008;83(4):531–41 10.1038/clpt.2008.3 18305452

[30] Xu X , Li B , Wang L Gender difference in smoking effects on adult pulmonary function . Eur Respir J. 1994;7(3):477–83 10.1183/09031936.94.07030477 8013605

